# Identifying factors that influence the use of pathogen genomics in Australia and New Zealand: a protocol

**DOI:** 10.3389/fpubh.2024.1426318

**Published:** 2024-10-23

**Authors:** James D. H. Ong, Tehzeeb Zulfiqar, Kathryn Glass, Martyn D. Kirk, Brad Astbury, Angeline Ferdinand

**Affiliations:** ^1^Evaluation and Implementation Science Unit, Centre for Health Policy, Melbourne School of Population and Global Health, The University of Melbourne, Melbourne, VIC, Australia; ^2^Department of Microbiology and Immunology, The Peter Doherty Institute for Infection and Immunity, The University of Melbourne, Melbourne, VIC, Australia; ^3^Department of Applied Epidemiology, National Centre for Epidemiology and Population Health, College of Health and Medicine, Australian National University, Canberra, ACT, Australia; ^4^Microbiological Diagnostic Unit Public Health Laboratory, The Peter Doherty Institute for Infection and Immunity, The University of Melbourne, Melbourne, VIC, Australia; ^5^Centre for Pathogen Genomics, University of Melbourne, Melbourne, VIC, Australia

**Keywords:** pathogen genomics, whole genome sequencing (WGS), policy use, infectious disease, qualitative comparative analysis (QCA), antimicrobial resistance

## Abstract

**Introduction:**

Pathogen genomics, where whole genome sequencing technologies are used to produce complete genomic sequences of pathogens, is being increasingly used for infectious disease surveillance and outbreak response. Although proof-of-concept studies have highlighted the viability of using pathogen genomics in public health, few studies have investigated how end-users utilize pathogen genomics in public health. We describe a protocol for a study that aims to identify key factors that influence the use of pathogen genomics to inform public health responses against infectious diseases in Australia and New Zealand.

**Methods:**

We will use qualitative comparative analysis (QCA), a case-oriented methodology that systematically compares and analyses multiple cases (or ‘units of analysis’), to identify multiple pathways leading to the use of pathogen genomics results in public health actions. As part of the process, we will develop a rubric to identify and define the use of pathogen genomics and individual factors affecting this process. Simultaneously, we will identify cases where pathogen genomics has been used in public health across Australia and New Zealand. Data for these cases will be collected from document review of publicly available and confidential documents and semi-structured interviews with technicians and end-users and summarized in a case report. These case reports will form the basis for scoring each case on the extent of the use of pathogen genomics data and the presence or absence of specific factors such as the ease of extracting essential information from pathogen genomics reports and perceptions toward pathogen genomics. Using the scores, cases will be analyzed using QCA techniques to identify pathways leading to the use of pathogen genomics data. These pathways will be interpreted alongside the cases to provide rich explanations of the use of pathogen genomics in public health.

**Discussion:**

This study will improve our understanding of the key factors that facilitate or hinder the use of pathogen genomics to inform public health authorities and end-users. These findings may inform ways to enhance the use of pathogen genomics data in public health.

## 1 Introduction

Public health responses against infectious disease typically involve conventional clinical microbiological testing to identify pathogens of interest, the results of which are incorporated into public health surveillance. This facilitates epidemiological investigations to identify likely sources of infection (1). More recently, public health laboratories have used whole genome sequencing (WGS) technologies to assist public health responses against infectious disease. WGS produces complete, accurate genomic sequences by simultaneously sequencing a large number of smaller DNA fragments in a sample which are then assembled into complete genomes via bioinformatics (2–4). Pathogen genomics applies WGS technologies to produce complete, accurate genomic sequences of pathogens (2–4). These sequences can be analyzed, compared and shared to describe the characteristics of the pathogen of interest and how it evolves. This enables public health teams to identify clusters, chains of transmission and infection sources that occur locally, between jurisdictions and across borders (2, 5). Hence, pathogen genomics data can complement epidemiological investigations and conventional clinical microbiology techniques to assist in surveillance and outbreak investigations (6–8) and to inform public health responses against infectious disease (2, 9, 10) and antimicrobial resistance (AMR) (11–13).

Public health authorities worldwide have steadily incorporated pathogen genomics into their public health systems for infectious disease surveillance. One early example is the US Food and Drug Administration’s GenomeTrakr, established in 2012 (14, 15). GenomeTrakr is a network of public health and university pathogen genomics laboratories that collect and share sequencing data from foodborne pathogens to identify potential foodborne-related outbreaks. This initiative is supported by public databases within the National Center for Biotechnology Information (NCBI) where sequencing data from US and internal pathogen genomics laboratories are openly shared. Similarly, the Gastrointestinal Bacterial Reference Unit in Public Health England began using pathogen genomics in 2014 to sequence *Salmonella* isolates for surveillance (16). Coming out of the COVID-19 pandemic, the World Health Organization is implementing the *Global Genomic Surveillance Strategy for Pathogens with Pandemic and Epidemic Potential, 2022–2032* (17). This global strategy will support countries to strengthen their capacity to conduct pathogen genomics to respond to pandemics and epidemics.

In Australia, pathogen genomics has been used to inform surveillance and outbreak responses against pathogens such as *Listeria monocytogenes* (18) and *Salmonella Typhimurium* (8). The COVID-19 pandemic raised the profile of pathogen genomics across Australia due to its numerous roles in informing public health responses. These include identifying SARS-CoV-2 variants (19), detecting SARS-CoV-2 genetic material from wastewater samples (20) and tracing the origins of COVID-19 clusters (21–23). Emerging from the COVID-19 pandemic, the Australian Pathogen Genomics (AusPathoGen) research program was established in 2021 to integrate pathogen genomics into public health systems across Australia and New Zealand to respond to infectious disease and AMR (24).

It is possible to evaluate the implementation of pathogen genomics programs in public health systems across Australia and globally. According to the Pathogen Genomics in Public HeAlth Surveillance Evaluation (PG-PHASE) framework by Ferdinand et al. (25), processes in pathogen genomics can be sorted into three phases:

1. The pre-analysis and analysis phase, where isolates are collected and sequenced;
2. The reporting and communication phase, where pathogen genomics data are communicated to end-users; and
3. The implementation phase, where pathogen genomics data are used to guide public health responses against infectious disease and AMR.

Evaluating pathogen genomics using this framework allows evaluators to understand how pathogen genomics data are generated, interpreted, and used. These evaluation results can then be considered to assess the overall implementation, utility, and effectiveness of pathogen genomics programs in public health. Despite the presence of this framework; however, there are few studies assessing the implementation, effectiveness and cost-effectiveness of public health pathogen genomics programs in real-time (11).

The use of pathogen genomics data is defined as the extent to which end-users such as policymakers and public health authorities utilize pathogen genomics data in surveillance and to inform public health responses against infectious disease. A variety of factors have been proposed that may affect how much and how effectively end-users such as policymakers and public health authorities utilize pathogen genomics data in public health (4, 25). These include how quickly pathogen genomics data can be delivered to end-users (11, 26), how easily end-users can extract essential information from pathogen genomics reports such as drug susceptibility and cluster details of isolates (27, 28) and what end-users think and know about pathogen genomics (29, 30). However, there is a lack of research identifying the most important factors affecting the use of pathogen genomics data among end-users such as policymakers and public health authorities.

This study aims to identify key factors influencing the use of pathogen genomics data by end-users such as policymakers and public health authorities to inform infectious disease surveillance and outbreak responses in Australia and New Zealand. This aim will be achieved by using qualitative comparative analysis (QCA), a methodology that compares cases or units of analysis to identify key factors that affect the probability of an outcome occurring. This protocol will describe how QCA will be conducted and presented in the study.

## 2 Methods and analysis

### 2.1 Study design

QCA is a technique that supports analysis of necessary and sufficient conditions leading to outcomes among a sample of cases. In this paper, a case, in the context of QCA, is a unit of analysis that is being compared such as an individual, event, program, site, organization, community or country. For this study, cases describe specific infectious disease surveillance programs or outbreak responses that involved pathogen genomics. In QCA, causality is viewed as complex and context-sensitive. The goal is not to identify a single explanatory model that fits the data best, but to reveal whether there are different combinations of conditions or pathways leading to the same outcome (31–33). These different pathways are produced by first summarizing and scoring a small to medium number of cases (typically 10–50 cases) (34). Boolean algebra is then used to convert the scores into pathways that are interpreted with reference to the individual cases (32, 33, 35). Referring the results back to the cases produces rich explanations of how different contextual factors interact to influence the use of pathogen genomics data among end-users.

QCA is an iterative process involving six steps (32):

1. Outcome and factor definition: A literature review of the topic is conducted to generate an initial rubric containing a definition and scoring criteria for each outcome and contextual factor.
2. Case selection: Cases are purposively selected to reflect a range of different outcomes, with variations in the presence or absence of individual factors among cases.
3. Case description: Data is collected from various sources such as documents and key informants to gain in-depth knowledge of each case.
4. Case summary: For each case, information from various data sources is summarized into one case report (36).
5. Data analysis: The case reports and rubric inform how each case will be scored on each outcome and factor (31, 37). These scores are placed in a data matrix which is converted to a truth table listing all possible factor combinations. The truth table is then analyzed via Boolean algebra to generate a set of pathways that independently lead to the outcome.
6. Results interpretation: The QCA results are interpreted alongside the cases to explain each pathway (37).

Initial results from the QCA may be fed back into earlier steps of QCA to test and refine the theoretical model of the use of pathogen genomics data. This can range from adjusting the definition and scoring criteria of specific outcomes and factors to collecting more information about a specific aspect of a case and re-scoring a particular outcome or factor of an individual case.

While QCA has been used extensively in other disciplines, it is only beginning to be applied in public health studies where variable-based analysis is the preferred approach among researchers (34). A key advantage of QCA in the present study is the potential to produce novel insights about what works for whom in different contexts rather than assuming there is ‘one right way’ to increase the use of pathogen genomics data among end-users. These end-users include, but are not limited to, policymakers, public health officers and clinicians.

### 2.2 Outcome and factor definition

For QCA, the outcome is the extent to which end-users such as policymakers and public health authorities use pathogen genomics data to inform surveillance or outbreak responses against an infectious disease. Factors are contextual elements that may affect the use of pathogen genomics data (31, 33) such as the timeliness of delivering pathogen genomics data to end-users (8, 26) and the ease of interpreting pathogen genomics reports (27, 28).

As part of the QCA, a rubric will be developed containing definitions and scores for each outcome and factor. Factors will be mainly derived from reviewing the Pathogen Genomics in Public HeAlth Surveillance Evaluation (PG-PHASE) Framework which describes how the impact of pathogen genomics in public health can be assessed (25). The Consolidated Framework for Implementation Research (CFIR), an implementation framework that lists contextual factors affecting implementation (38), will also be reviewed to identify potential factors affecting the use of pathogen genomics data. Lastly, genomic epidemiologists and members of the AusPathoGen evaluation team will be consulted to identify potential factors affecting the use of pathogen genomics data.

These factors, along with the outcome, will be placed in a rubric. The rubric will be further refined by reviewing pathogen genomics guidelines and studies to inform the development of definitions and preliminary scores for each outcome and factor. Scores for each outcome and factor will be defined using a four-value fuzzy set involving values of 0, 0.33, 0.67 and 1 (39). This scoring set will be used as it accounts for situations where an outcome or factor might be more present than absent, and vice versa (39, 40). The rubric will be tested on a sample of overseas cases and, in consultation with the AusPathoGen evaluation team, will be further refined in steps 3 to 5 of the QCA.

A preliminary rubric for the study can be found in Supplementary Table 1.

### 2.3 Case selection

Cases will be chosen for QCA using the following selection criteria:

1. Pathogen genomics was used in a surveillance program or outbreak response against an infectious disease in Australia and New Zealand in 2018 and beyond;
2. Samples of the pathogen were extracted and run through a WGS platform, not solely relying on retrospective genomic data that had already been sequenced; and
3. There was an attempt to inform end-users about pathogen genomics data for the purposes of disease prevention and control.

Potential cases will initially be identified from a literature search of peer-reviewed articles over the PubMed, EMBASE and CINAHL databases. For each database, after filtering articles to 2018 and beyond and Australia and New Zealand, titles and abstracts will be reviewed to make a preliminary assessment of the article’s relevance against the selection criteria. Articles that pass the preliminary assessment will be downloaded and read to further assess their fit against the selection criteria. Articles that satisfy the selection criteria will be cataloged with details of the surveillance system or outbreak response such as the pathogen sequenced, the year the case took place and the outcome and policy implications from using pathogen genomics data. A meeting will be arranged with the contact person of the peer-reviewed article to learn more about the case and to assess whether the case would be suitable for the study. Further cases of the use of pathogen genomics data will also be added to the catalog from consultations with members of the AusPathoGen evaluation team and genomic and public health epidemiologists from different jurisdictions of Australia and New Zealand.

Cases from both approaches will be pooled, with at least 10 cases selected for QCA to account for variations in outcomes and factors across cases and time to explore each case in detail (36). These cases, coming from different jurisdictions in Australia and New Zealand, will reflect the varying extent to which pathogen genomics data were used, from not being used at all to playing an influential role in directing public health responses against infectious disease. This is consistent with the requirement in QCA to select cases that exhibit a range of different conditions and outcomes to produce robust findings on alternative pathways to successful the use of pathogen genomics data (31).

### 2.4 Case description

For each case, data will be collected via document review and semi-structured interviews. Initially, documents will be collected and reviewed to understand the context of the case and to extract relevant information on the outcome and factors. Depending on the information present in the documents, semi-structured interviews will be organized with key informants to confirm existing information and, where required, to collect additional evidence for the QCA.

#### 2.4.1 Document review

Peer-reviewed articles relating to the case will be identified from a literature search over the PubMed, EMBASE and CINAHL databases. Documents and articles relating to the case that are publicly available will be sourced via a Google search, while those that are not publicly available will be identified in consultation with key informants. Documents include, but are not limited to, pathogen genomics reports, laboratory reports and meeting minutes.

#### 2.4.2 Semi-structured interviews

Key informants of the case who processed and presented pathogen genomics data such as public health laboratory staff and genomic epidemiologists, as well as end-users of pathogen genomics data such as public health specialists and decision makers, will be contacted via email and invited to an interview. Within the invitation, participants will receive an information sheet about the study and a consent form. Should they consent to participate, a time and date for the interview will be organized and a list of interview questions provided to facilitate preparation. Interview participants will be asked questions relating to the details of the case, the extent to which end-users used pathogen genomics data to inform their public health policies, and decisions and factors influencing the use of pathogen genomics data. Additional questions may be asked to confirm or collect information on specific factors of the use of pathogen genomics data.

Interviews will be conducted either in-person or on Zoom and, with the interviewee’s consent, recorded while notes are taken. Interview audio recordings will be transcribed and key informants and end-users will be given the opportunity to review the transcript and make corrections. Once the transcript is finalized, it will be used for data analysis alongside the interview notes.

### 2.5 Case summary

Documents and interview transcripts for each case will be coded in NVivo 12 (QSR International, Denver, Colorado), following the methods of Miles et al. (41). Briefly, this involves reading each document and interview transcript in detail and annotating them with researcher memos to familiarize and reflect on each piece of data. For each case, documents and interview transcripts will undergo two cycles of coding, with the first cycle coding parts of documents or interview transcripts and the second cycle sorting the codes into outcomes and factors influencing the use of pathogen genomics data. The code groupings will be used to write a case report summarizing the circumstances, outcome and factors of use of pathogen in the case and providing a researcher interpretation of the case.

### 2.6 Data analysis

**Figure 1:**
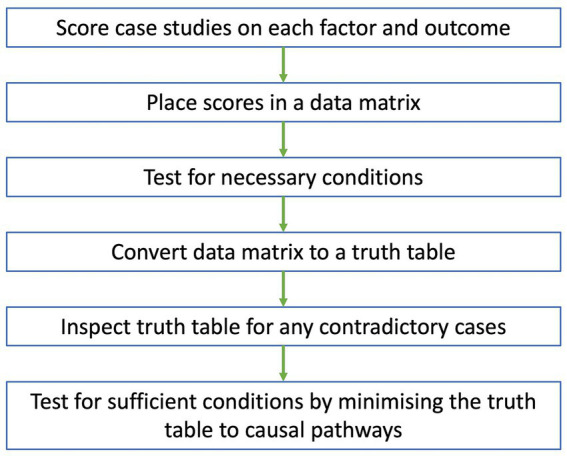
Flow chart of data analysis for QCA. Case studies are scored on each factor and outcome, with scores placed in a data matrix. A test for necessary conditions is conducted to identify any necessary conditions from the data matrix. The data matrix is then converted to a truth table which is inspected for any contradictory cases. After resolving all contradictions, a test for sufficient conditions is conducted, where the truth table is minimized to a set of causal pathways that independently lead to the outcome.

Data analysis in QCA will be conducted according to the flow chart in Figure 1. The scoring of cases for each factor and outcome will be conducted by two authors. Initially, they will meet to agree on a process for scoring cases. The researchers will then independently score all cases on each factor and outcome using information provided from the case reports and rubric. The scores, along with the justification behind them, will be placed in a scoring worksheet. Key informants and end-users will be consulted to ensure the scores are reflective of what happened in each case. After scoring all cases, the researchers will meet again to discuss and moderate differences in scores.

Once a consensus is reached for all scores, they will be placed in a data matrix and imported as a CSV file into fsQCA software (University of California, Irvine, California). Initially, the data matrix will be explored by generating XY plots between the outcome and single factors along the y- and x-axes, respectively. If all cases fall below the main diagonal of the XY plot, then the factor might be necessary for the outcome, meaning that the factor must be present for the outcome to occur (33). This will be confirmed by conducting a test for necessary conditions. Any factors identified as necessary under the test may be removed from the data matrix and considered separately from other results.

After determining the best approach for necessary factors, the final data matrix will be converted to a truth table, where cases are grouped into different combinations of factor scores. After deleting rows that do not have any cases, the truth table will be sorted in descending order of raw consistency, defined as the extent that a combination of factors leads to the outcome (37). Based on a raw consistency cut-off point of 0.75 or above (42), each row of the truth table will be numbered 1 or 0 on the outcome column representing the presence or absence of the use of pathogen genomics data, respectively. The truth table will then be inspected to identify any contradictory cases that have an identical combination of factor scores but different outcome scores (31, 32, 34). Any contradictory cases will be resolved using the strategies from Rihoux and De Meur (43). This may include adding or removing factors from the data matrix, re-examining the contradictory cases to re-scores factors and outcomes or recoding the outcome of all contradictory cases.

If an outcome will always occur when a condition or a combination of conditions are present, then the factor or factor combination is sufficient for the outcome (33). Sufficient conditions will be identified via a test for sufficient conditions involving Boolean minimization, where the truth table is minimized to a set of pathways that independently lead to the outcome (33). Assumptions can be added based on the literature of the use of pathogen genomics data to simplify the pathways (33, 39). Solution and raw coverage scores will be calculated, representing the proportion of cases that are included in the overall set and each pathway respectively, to assess the strength and relevance of the findings (33).

### 2.7 Results interpretation and presentation

The QCA results will be interpreted alongside the cases. This will be done by checking each pathway against case reports, documents and interview transcripts of cases in the pathway to confirm links between factors and outcomes, to explore cross-case patterns and to produce generalizations that can be applied to similar cases (32). QCA interpretation may also involve going back to outcome and factor definition to refine definitions and scoring criteria, case selection to select more cases, case description and summary to collect and summarize new information from existing cases and/or data analysis to re-score cases (39). Changes to any step of QCA will be noted and reported to ensure transparency of the model testing and refinement process.

QCA results will be presented and visualized using different examples from Rubinson (44). These include, but are not limited to, Boolean expressions, mathematical descriptions of pathways leading to the presence or absence of an outcome; consistency/coverage tables, a table of necessary and sufficient pathways along with their consistency and coverage scores; and Fiss charts, a visualization of different pathways showing the absence or presence of individual factors (44).

## 3 Discussion

Retrospective, proof-of-concept studies in a research setting have highlighted the potential of pathogen genomics to inform surveillance and public health responses against infectious disease and AMR (5, 45, 46). However, there are few implementation studies describing the incorporation of pathogen genomics into public health systems to assist in public health responses against infectious disease and AMR. In particular, there is a dearth of studies looking at how pathogen genomics data are used to inform public health policies to control infectious disease and the key factors influencing this process (4, 25).

This study will bridge the implementation gap from proof-of-concept studies to consider processes that affect the use of pathogen genomics data in public health settings. Using QCA, the study will harness cases across various places and contexts to identify key contextual factors that affect how much end-users use pathogen genomics data to inform public health responses against infectious disease and AMR. The main strength of QCA is that it can simplify cases to a set of pathways that independently lead to the use of pathogen genomics data (35). These pathways are moderately generalizable in that key factors can be drawn from them to explain the use of pathogen genomics data in other countries, particularly low- and middle-income countries (LMICs) that are establishing their own pathogen genomics-based infectious disease surveillance systems. Despite LMICs facing funding and resource constraints, factors such as pathogen genomics literacy and collaboration between pathogen genomics experts and end-users can still influence the use of pathogen genomics data in LMICs (47, 48). Hence, understanding the key factors that facilitate the use of pathogen genomics data can help LMICs find the best way to direct their limited resources to implement pathogen genomics in-country. At the same time, by contextualizing the pathways to the cases, we can generate rich explanations of how certain contextual factors affect the use of pathogen genomics data. Taken together, this can help program managers promote the use of pathogen genomics data among end-users in their country, depending on whether certain factors are present or absent. A potential limitation of using QCA in the study is that it relies on a large amount of information for each case. This is required to accurately score all cases on each outcome and factor to complete the data matrix, allowing conclusions to be drawn. Any factor that does not have a score for a specific case presents the difficult decision of dropping either the case or factor (34). Additionally, QCA requires a large amount of background research to identify factors that are estimated to have the biggest impact on the use of pathogen genomics data and appropriate cases to study (31, 36). This limits the number of factors and cases that can be investigated. Both limitations may restrict the range of findings from QCA that could be drawn to explain the use of pathogen genomics data by end-users.

Nevertheless, this study will seek to improve our understanding of how end-users use pathogen genomics data to guide public health responses against infectious disease and AMR. For the first time, the study will identify key factors that facilitate or hinder the use of pathogen genomics data among end-users. These findings will recommend ways to enhance the reporting, communication and utilization of pathogen genomics data to improve public health responses against infectious disease. In particular, the findings can be used to inform planning and implementation of programs that aim to promote the use of pathogen genomics data among end-users. This will hopefully increase the use of pathogen genomics data among end-users in public health policy, facilitating tailored and more timely responses against infectious disease and AMR that may help control an outbreak or pandemic sooner.

## 4 Ethics and dissemination

### 4.1 Ethics statement

This study received formal ethics approval as part of the ethics application of the AusPathoGen evaluation from Australian National University’s Science and Medical Delegated Ethical Review Committee (protocol 2022/407).

### 4.2 Dissemination

Findings from the QCA will be initially described in reports that are tailored to different audiences. These reports will be targeted to public health laboratories and units in Australia and New Zealand to find ways to improve utilization of pathogen genomics data in public health. The reports will also be forwarded to the AusPathoGen executive group to plan programs that aim to enhance the use of pathogen genomics data among end-users.

Externally, key findings from the study will be published in academic journals and presented in meetings and conferences for technicians and policymakers in the pathogen genomics field. Progress and findings from the QCA study will also be summarized in the AusPathoGen newsletter that is distributed to internal AusPathoGen staff and external staff in public health laboratories and units and state and federal governments.

## Author’s note

For information on AusPathoGen, visit https://www.auspathogen.org.au.
