# Supplementary material for: Identifying factors that influence the use of pathogen genomics in Australia and New Zealand: a protocol
Source: Front Public Health. 2024 Oct 23;12:1426318. doi: 10.3389/fpubh.2024.1426318 (PMC11537980; doi:10.3389/fpubh.2024.1426318)
Supplement: Supplementary file 1 [file Table_1.DOCX]

Supplementary Material

**Supplementary Table 1**: Preliminary rubric for QCA

| **Factor name** | **Definition** | **Reference** | **Preliminary ratings** |
| --- | --- | --- | --- |
| Outcome: Extent of use of pathogen genomics results | To what extent do policymakers or public health authorities use pathogen genomics results to inform the surveillance or outbreak response against an infectious disease? | 1-3 | - **0**: pathogen genomics did not contribute to the surveillance program or outbreak response against infectious disease - **0.33**: pathogen genomics only played a minor role to the surveillance program or outbreak response against infectious disease; it is overshadowed by external factors and/or laboratory and/or epidemiological data - **0.67**: pathogen genomics has clearly contributed to the surveillance program or outbreak response against infectious disease, rising above external factors and complementing laboratory and/or epidemiological data - **1**: pathogen genomics played an essential role in informing the surveillance program or outbreak response (i.e., without pathogen genomics, the public health response would never have happened) |
| Factor 1: timeliness | How quickly can pathogen genomics results be delivered to policymakers and public health authorities to inform public health responses against infectious disease? | 1,4-7 | - **0**: pathogen genomics results were delivered too late to inform public health responses - **0.33**: there is a delay in pathogen genomics results being delivered, but they still informed public health responses - **0.67**: pathogen genomics results were delivered in a timely manner to inform public health responses - **1**: pathogen genomics results are delivered in real time with new data continually updating results, allowing an immediate response to be set up |
| Factor 2: Links to other data sources | To what extent do pathogen genomics results complement laboratory or epidemiological data from the surveillance program or outbreak response? | 4-6,8-11 | - **0**: pathogen genomics results did not complement or conflicted with laboratory and epidemiological data. - **0.33**: pathogen genomics results only partially complemented laboratory or epidemiological data - **0.67**: pathogen genomics results completely complements laboratory or epidemiological data - **1**: pathogen genomics results completely complements both laboratory and epidemiological data |
| Factor 3: Novel data from pathogen genomics | How much new information did pathogen genomics reveal to policymakers regarding the surveillance or outbreak response of infectious disease? | 3 | - **0**: pathogen genomics did not produce any new information for the policymaker - **0.33**: pathogen genomics produced some new information that confirmed a public health action that already took place - **0.67**: pathogen genomics produced some new information that confirmed the policymaker’s decision on the surveillance program or outbreak response - **1**: pathogen genomics produced a lot of new and/or surprising information that altered public health responses against infectious disease |
| Factor 4: Ease of interpreting reports | How easy can policymakers interpret pathogen genomics reports and extract essential information to inform public health responses? | 1,2,5,8,12-14 | Five design guidelines (12):   1. Structure information such that it mimics a stakeholder’s workflow: data and information are ordered according to what the reader needs 2. Use emphasis carefully: only use emphasis to highlight important data/information 3. Present dense information in a careful and structured manner: have a one-page summary and a multi-page appendix (13) 4. Use words precisely: ensure terminology can be understood by technicians, clinicians and policymakers 5. If using images, do so judiciously  - **0**: None of the design guidelines were fulfilled - **0.33**: 1-2 out of 5 design guidelines were fulfilled - **0.67**: 3-4 out of 5 design guidelines were fulfilled - **1**: all 5 design guidelines were fulfilled |
| Factor 5: Attitude towards pathogen genomics | What do policymakers think about how pathogen genomics can inform public health responses against infectious disease? | 1,11,15,16 | - **0**: policymakers oppose the use of pathogen genomics to inform public health responses - **0.33**: policymakers are either neutral or unsure about the use of pathogen genomics to inform public health responses - **0.67**: policymakers have a positive view about the use of pathogen genomics to inform public health responses - **1**: policymakers are not only enthusiastic about the use of pathogen genomics to inform public health responses, but they are also championing it in their department |
| Factor 6: Pathogen genomics literacy among policymakers | How much do policymakers know about pathogen genomics and interpreting pathogen genomics reports to inform public health responses? | 4,5,8,17 | - **0**: policymakers do not understand how pathogen genomics works (i.e., they cannot use pathogen genomics results to inform public health responses) - **0.33**: policymakers have a basic understanding of how pathogen genomics works (i.e., they can pick key results of pathogen genomics reports to inform public health responses) - **0.67**: policymakers have a competent level of understanding of how pathogen genomics works (i.e., they understand the strengths and limitations of pathogen genomics and how pathogen genomics results complement other data sources) - **1**: policymakers have an advanced understanding of how pathogen genomics works (i.e., the technology behind pathogen genomics) |
| Factor 7: Data quality | How confident can end-users rely on pathogen genomics results to inform public health responses against infectious disease? | 6,8,18 | - **0**: data quality is so poor that pathogen genomics results could not be relied on to inform public health responses - **0.33**: data quality is acceptable, but a lot of data is missing - **0.67**: data quality is acceptable, and there is enough data to inform public health responses - **1**: data quality is very high and is almost or fully complete |
| Factor 8: Communication channels | How open are communication channels between policymakers and pathogen genomics experts? | 1,5,6,8 | - **0**: communication channels are completely closed, policymakers do not speak to pathogen genomics experts - **0.33**: pathogen genomics experts are only consulted when making public health decisions; they are not invited to meetings with policymakers and not involved in the decision-making process - **0.67**: pathogen genomics experts are invited to meetings with policymakers, but are not involved in the decision-making process - **1**: pathogen genomics experts are invited to meetings with policymakers AND are involved in the decision-making process |

## References

1. Ferdinand AS, Kelaher M, Lane CR, et al. An implementation science approach to evaluating pathogen whole genome sequencing in public health. *Genome Med*. 2021;13(1):121. doi:10.1186/s13073-021-00934-7

2. Hilt EE, Ferrieri P. Next generation and other sequencing technologies in diagnostic microbiology and infectious diseases. *Genes*. 2022;13(9). doi:10.3390/genes13091566

3. Ledermann S. Exploring the necessary conditions for evaluation use in program change. *American Journal of Evaluation*. 2011;33(2):159-178. doi:10.1177/1098214011411573

4. Beukers AG, Jenkins F, van Hal SJ. Centralised or localised pathogen whole genome sequencing: Lessons learnt from implementation in a clinical diagnostic laboratory. *Front Cell Infect Microbiol*. 2021;11:636290. doi:10.3389/fcimb.2021.636290

5. Ford L, Carter GP, Wang Q, et al. Incorporating whole-genome sequencing into public health surveillance: Lessons from prospective sequencing of Salmonella Typhimurium in Australia. *Foodborne Pathogens and Disease*. 2018;15(3):161-167. doi:10.1089/fpd.2017.2352

6. Stevens EL, Carleton HA, Beal J, et al. Use of whole genome sequencing by the federal interagency collaboration for genomics for food and feed safety in the United States. *J Food Prot*. 2022;85(5):755-772. doi:10.4315/jfp-21-437

7. Waddington C, Carey ME, Boinett CJ, Higginson E, Veeraraghavan B, Baker S. Exploiting genomics to mitigate the public health impact of antimicrobial resistance. *Genome Med*. 2022;14(1):15. doi:10.1186/s13073-022-01020-2

8. Black A, MacCannell DR, Sibley TR, Bedford T. Ten recommendations for supporting open pathogen genomic analysis in public health. *Nat Med*. 2020;26(6):832-841. doi:10.1038/s41591-020-0935-z

9. Brown B, Allard M, Bazaco MC, Blankenship J, Minor T. An economic evaluation of the whole genome sequencing source tracking program in the U.S. *PLoS One*. 2021;16(10):e0258262. doi:10.1371/journal.pone.0258262

10. Leekitcharoenphon P, Nielsen EM, Kaas RS, Lund O, Aarestrup FM. Evaluation of whole genome sequencing for outbreak detection of Salmonella enterica. *PLOS ONE*. 2014;9(2):e87991. doi:10.1371/journal.pone.0087991

11. Van Goethem N, Descamps T, Devleesschauwer B, et al. Status and potential of bacterial genomics for public health practice: A scoping review. *Implement Sci*. 2019;14(1):79. doi:10.1186/s13012-019-0930-2

12. Crisan A, McKee G, Munzner T, Gardy JL. Evidence-based design and evaluation of a whole genome sequencing clinical report for the reference microbiology laboratory. *PeerJ*. 2018;6:e4218. doi:10.7717/peerj.4218

13. Tornheim JA, Starks AM, Rodwell TC, et al. Building the framework for standardized clinical laboratory reporting of next-generation sequencing data for resistance-associated mutations in Mycobacterium tuberculosis complex. *Clinical Infectious Diseases*. 2019;69(9):1631-1633. doi:10.1093/cid/ciz219

14. Valenstein PN. Formatting patholgy reports: Applying four design principles to improve communication and patient safety. *Arch Pathol Lab Med*. 2008;132(1):84-94.

15. Degeling C, Johnson J, Gilbert GL. Perspectives of Australian policy-makers on the potential benefits and risks of technologically enhanced communicable disease surveillance - a modified Delphi survey. *Health Research Policy & Systems*. 2019;17(1). doi:10.1186/s12961-019-0440-3

16. Kwong JC, McCallum N, Sintchenko V, Howden BP. Whole genome sequencing in clinical and public health microbiology. *Pathology*. 2015;47(3):199-210. doi:10.1097/pat.0000000000000235

17. Van Goethem N, Struelens MJ, De Keersmaecker SCJ, et al. Perceived utility and feasibility of pathogen genomics for public health practice: a survey among public health professionals working in the field of infectious diseases, Belgium, 2019. *BMC public health*. 2020;20(1):1318. doi:10.1186/s12889-020-09428-4

18. Jagadeesan B, Gerner-Smidt P, Allard MW, et al. The use of next generation sequencing for improving food safety: Translation into practice. *Food Microbiol*. 2019;79:96-115. doi:10.1016/j.fm.2018.11.005
